# Impairment of Ceramide-Mediated Endothelial Instant Membrane Resealing During Diabetes Mellitus

**DOI:** 10.3389/fphys.2022.910339

**Published:** 2022-07-06

**Authors:** Yang Chen, Guangbi Li, Owais M. Bhat, Xiang Li, Yang Zhang, Pin-Lan Li

**Affiliations:** ^1^ School of Chinese Materia Medica, Guangzhou University of Chinese Medicine, Guangzhou, China; ^2^ Department of Pharmacology and Toxicology, School of Medicine, Virginia Commonwealth University, Richmond, VA, United States; ^3^ Department of Pharmacological and Pharmaceutical Sciences, College of Pharmacy, University of Houston, Houston, TX, United States

**Keywords:** membrane repair, sphingolipid, endothelial dysfunction, annexin, hyperglycemia

## Abstract

Recent studies have indicated that instant cell membrane resealing (ICMR) controls the activation of NOD-like receptor pyrin domain containing 3 (Nlrp3) inflammasomes in endothelial cells, thereby initiating and promoting vascular inflammation. It remains unknown whether this impaired ICMR occurs under diabetic condition or hyperglycemia contributing to endothelial dysfunction leading to vascular inflammation, a hallmark of diabetic vascular injury. The present study aims to examine whether ICMR occurs during in control and diabetic mice and to explore related molecular mechanisms associated with acid sphingomyelinase (ASM)-mediated ceramide production. Using confocal microscopy, we demonstrated that mouse aortic endothelial cells (MAECs) exposed to high glucose levels exhibited much more retarded ICMR after laser-induced membrane injury, compared to that in control cells. The high glucose-induced impairment of membrane resealing in MAECs was prevented when these cells were pretreated with sphingomyelin or C24-ceramide. Mechanistically, high glucose treatment decreased association of membrane ceramide with annexin A5, an essential element of membrane repair machinery. Consistently, the association of ceramide with annexin A5 was significantly reduced in the coronary arterial endothelium of mice with streptozotocin-induced diabetes mellitus compared to that in non-diabetic control mice. Moreover, a marked reduction of the association of ceramide with annexin A5 was observed in coronary arterial endothelium of ASM knockout mice regardless of their diabetic status. Lastly, high glucose treatment or ASM gene deletion substantially impaired ICMR in coronary arterial endothelium of mice receiving membrane puncturing agents. Collectively, our data suggest that ceramide-mediated ICMR in vascular endothelial cells is impaired during diabetes mellitus due to dissociation of ceramide with annexin A5 and ASM play a critical role in this ICMR.

## Introduction

Recent studies demonstrate that vascular complications are the major causes of disability and death in patients with diabetes mellitus (Kandula et al., 2016; Zimmet et al., 2016; Takase et al., 2017). It has been well established that endothelial dysfunction is an early onset of diabetes-associated vascular diseases that contributes to vascular inflammation and injury (Liye et al., 2011; Chen et al., 2016a). However, the precise mechanism initiating endothelial cell dysfunction and injury remains largely unknown, particularly, at the early stage of diabetes mellitus. Hyperglycemia is considered as one of the main factors for the development of diabetic vasculopathy (Laight et al., 1999). High glucose causes direct injurious effects on endothelial cells including production of reactive oxygen species (ROS), disruption of calcium homeostasis, induction of apoptosis, and activation of Nlrp3 inflammasomes (Mattson et al., 2000; Yu et al., 2011; Chen et al., 2016a). These high glucose-induced injurious effects are implicated in endothelial cell activation, inflammation, or injury leading to endothelial dysfunction in diabetes mellitus. However, little is known about the effect of high glucose on the plasma membrane repair machinery in endothelial cells. It is imperative to investigate whether hyperglycemia causes endothelial cells more vulnerable to membranous injury and thereby contributes to endothelial cell injury.

Plasma membrane is a biological barrier that separates the interior of all cells from the outside environment. Plasma membrane disruption is a naturally occurring phenomenon in mechanically active tissues and a common form of cell injury under physiological and pathological conditions (McNeil and Steinhardt, 2003). Recent studies have indicated instant cell membrane resealing (ICMR) machinery during injury is an important adaptive mechanism to repair membrane and essential for cell survival and function (McNeil, 2002; Abreu-Blanco et al., 2012). In case that plasma membrane resealing machinery is insufficient to instantly repair the membrane disruption, the cells may function abnormally or die due to the loss of cytoplasm and to the entry of extracellular molecules (Abreu-Blanco et al., 2012). Recent studies have demonstrated that ceramide plays a critical role in cell membrane repair during cell injury (Draeger et al., 2014). Acid sphingomyelinase (ASM), a lysosomal hydrolase that metabolizes sphingomyelin into ceramide and phosphocholine, is translocated onto the plasma membrane through lysosome trafficking and fusion upon stimulation (Jin et al., 2008; Li et al., 2013). Membrane rafts (MRs; also known as lipid rafts) are dynamic assemblies of cholesterol, lipids with saturated acyl chains, such as sphingomyelin and glycosphingolipids, in the exoplasmic leaflet of the membrane bilayer, and cholesterol in the inner leaflet (Zhang Y. et al., 2009). Ceramides spontaneously fuse small MRs into large ceramide-enriched membrane microdomains that serve as signaling platforms to reorganize and cluster receptors and signaling molecules (Zhang Y. et al., 2009). In this respective, ASM has been shown to promote MR clustering and participate in the control of cell membrane resealing (Miller et al., 2015). It has also been reported that high glucose can modulate ceramide and MR-mediated signaling pathways (Somanath et al., 2009; Wang et al., 2013). Therefore, it is intriguing to know whether high glucose impairs ceramide-mediated instant membrane repair in endothelial cells.

In the present study, we first determined whether the membrane resealing following laser-induced membrane injury is impaired under high glucose condition and explored the role of ASM-ceramide pathway in this process using primarily cultured endothelial cells. Then, we determined whether the endothelial cells in the coronary arteries of diabetic mice fails to repair injured membranes upon intravenously administrated membrane puncturing agents.

## Materials and Methods

### Isolation of ECs From Mouse Aorta

The mouse aortic endothelial cells (MAECs) were primarily cultured from ASM^+/+^ (WT) or ASM^−/−^ (ASMKO) mice. WT and ASMKO mice (3 weeks of age, male) were bred from breeding pairs from the Jackson Laboratory (Bar Harbor, ME, United States). The ASMKO mice were originally developed as reported (Horinouchi et al., 1995). The mouse genotyping was performed using primers ASM-PA 1-2: 5′-CGA GAC TGT TGC CAG ACA TC-3´; ASM-PA 2-2: 5′-GGC TAC CCG TGA TAT TGC TG-3´; ASM-PS-2: 5′-AGC CGT GTC CTC TTC CTT AC-3′ as described (Horinouchi et al., 1995; Boini et al., 2011; Li et al., 2013; Zhang et al., 2018). All protocols were approved by the Institutional Animal Care and Use Committee of Virginia Commonwealth University (#A3281-01). Mice were anesthetized with an intra-peritoneal injection of pentobarbital sodium (300 mg/kg body weight) plus 500 U heparin. The aorta and branches of the heart artery were removed and placed in Ca^2+^-free phosphate-buffered saline (PBS). Periadventitial fats and connective tissues around the vessels were carefully cleaned under a dissecting microscope using forceps and iris scissors. Matrigel (BD Biosciences, San Jose, CA) was added to 24-well plates (about 250 μl in each well) and polymerized at 37°C for 30 min. The tissue was cut into 8–10 small pieces and opened longitudinally. These pieces were placed with the intima side down on the Matrigel in the specific endothelial cell culture wells (4–5 pieces in each well). Next, a small amount of culture media was added to keep the explants moist but not submerged. The explants were placed in an incubator at 37°C in a 5% CO_2_ atmosphere, and cells migrated from the aortic segments. After 5–7 days, the aortic pieces were removed, and MAECs were maintained in Complete Mouse Endothelial Cell Medium (MECM) (Cell biologics, Chicago, IL, United States), supplemented with 10% fetal bovine serum (FBS), 0.1% VEGF, 0.1% ECGS, 0.1% EGF, 0.1% hydrocortisone, 1% l-glutamine, 1% antibiotic-antimycotic Solution. The cells were cultured in a humidified incubator a mixture at 37°C with 5% CO_2_ and 95% air. Cells were passaged by trypsinization (Tripsin/EDTA, Sigma, St. Louis, MO, United States), followed by stopping the reaction in the Complete Mouse Endothelial Cell Medium. MAECs were pretreated with C-24 ceramide or sphingomyelin (Cayman, Ann Arbor, MI, United States) or treated as described.

### Analysis of Plasma Membrane Injury in MAECs

Plasma membrane injury in MAECs was assayed by measuring laser-wounding-induced FM-143 uptake as previously described (Bansal et al., 2003). Briefly, the cells were cultured in the record chamber and membrane damage was induced in the presence of FM-143 dye (2.4 μM; Molecular Probes) with a two-photon confocal laser-scanning microscope (LSM 510; Zeiss) coupled to a 10-W Argon/Ti: 6 sapphire laser. To induce injury, a 4 μm × 4 μm area of the membrane on the surface of the MAECs was irradiated at full power for 6.4 s at t = 20 s. Images were captured beginning 20 s before (t = 0) and for 5 min after the irradiation at 10-s intervals and the fluorescence intensity at the site of the damage was measured with the Zeiss LSM 510 imaging software. MAECs that had no membrane resealing showed dye filling at the wound site over the entire course of the experiment, whereas dye influx halted within 2 min for the membrane that resealed under the experimental conditions. FM-143 dye uptake was quantified by determining dF/Fo [= (Ft−Fo)/Fo] (Ft: the mean fluorescence intensity at time t; Fo: initial fluorescence intensity) after wounding within a 40 μm × 40 μm ROI. Metafluor imaging and analysis software was used to acquire, digitize and store the images for off-line processing and statistical analysis.

### Flow Cytometric Analysis

The surface expression of annexin A5 and ceramide in MAECs was analyzed by flow cytometry. In brief, MAECs were harvested and washed with PBS 0.2% Tween 20 and incubated with anti-annexin A5 antibody (1:200, Abcam, Cambridge, MA, United States) and anti-ceramide antibody (1:20, MID 15B4, Enzo, Ann Arbor, MI, United States) for 30 min on ice. The cells were fixed with paraformaldehyde, washed with PBS 0.2% Tween 20, and resuspended in ice cold PBS with 10% FCS and 1% sodium azide. Cells were analyzed through a Guava Easycyte Mini Flow Cytometry System (Guava Technologies, Hayward, CA, United States) using Guava acquisition and analysis software.

### Immunoprecipitation

Cells were washed twice with PBS and scraped in lysis buffer containing a protease inhibitor cocktail (Roche), 30 mM Tris-HCl, 150 mM NaCl, 2 mM EDTA, 1% Triton X-100, 10% glycerol. The nuclei and cell debris of the lysates were spun down (5,000 ×g for 5 min at 4°C) and the supernatant (termed homogenate) containing the microsome and cytosolic fractions were collected. Microsomes and cytosols were separated by a differential centrifugation of the homogenate at 10,000 g for 20 min and then at 100,000 g for 90 min. The pellet (microsomes) was resuspended in lysis buffer and prepared for immunoprecipitation as we previously described (Zhang et al., 2011). Then, lysate samples were centrifuged, and the supernatants were precleared by incubation with Protein A/G PLUS-agarose beads (Santa Cruz Biotechnology) at 4°C. The precleared supernatant was incubated 7 with 2 μg of antibody against ceramide for 4 h at 4°C. Beads were added for an additional 1 h, and then immunoprecipitates were collected by centrifugation at 1,000 × g for 5 min and washed three times with immunoprecipitation lysis buffer. The pellet was resuspended in 2 × SDS sample buffer, boiled. Samples were run into SDS-PAGE gel, transferred into PVDF membrane and blocked with skimmed milk. The membranes were probed with anti-annexin A5 antibody (1:200, Abcam, Cambridge, MA, United States) overnight at 4°C followed by incubation with secondary antibody conjugated to horseradish peroxidase (1:5,000, Santa Cruz, Dallas, TX, United States). The immunoreactive bands were enhanced by chemiluminescence reagents (Pierce, Rockford, IL, United States) and imaged on Kodak Omat film. β-actin reporting was used as a loading control. The intensity of the bands was quantified by densitometry.

### Confocal Microscopy Analysis of Annexin A5 and Ceramide in Coronary Arterial Intima of Mice

Confocal immunofluorescence analysis was performed to detect co-localization of different molecules in the coronary arterial endothelium as described (Wei et al., 2013; Chen et al., 2015b). In brief, the mouse hearts were frozen in Tissue-Tek OCT and cut by cryostat into 10-μm sections and mounted on Superfrost/Plus slides. After fixation with acetone, the frozen section slides were incubated with anti-vWF antibody (1:500, Abcam) and anti-annexin A5 (1:200, Abcam) or anti-ceramide antibody (1:20, Enzo) overnight at 4oC. After incubation with primary antibodies, the slides were washed and labeled with corresponding Alexa Fluor-488 and Alexa Fluor-555 conjugated secondary antibodies (Invitrogen). The slices were washed, mounted, and visualized through sequentially scanning on an Olympus laser scanning confocal microscope (Fluoview FV1000, Olympus, Japan). Co-localization was analyzed by image Pro Plus software, and the co-localization coefficient was represented by Pearson’s correlation coefficient.

### *In vivo* Membrane Repair Assay

To determine whether the plasma membrane of endothelial cells fails to repair *in vivo*, we performed a sequential labeling assay using YOYO-1, a membrane-impermeable dye (green florescence), and propidium iodide (PI). The diabetic mice were first administered with YOYO-1 (1.2 μl/g) through inguinal veins. Two hours following YOYO-1 injection, the mice were similarly administered with PI (7 μl/g) or *Lactobacillus* casei cell wall fragments (LCWE, 1 μg/g) together with PI (7 μl/g). The 8 cells that are repaired from LCWE-induced membrane injury are only labeled with YOYO-1. The cells with persistent plasmalemma damages are labeled with both YOYO-1^+^ and PI^+^ when is LCWE added. After 2 h, collect the tissue and cut the frozen slices immediately. Then, the slides were mounted and visualized through sequentially scanning on an Olympus laser scanning confocal microscope (Fluoview FV1000, Olympus, Japan). Co-localization was analyzed by Image Pro Plus software, and the co-localization coefficient was represented by Pearson’s correlation coefficient.

### Statistics

Data are presented as means ± SE. Significant differences between and within multiple groups were examined using ANOVA for repeated measures, followed by Duncan’s multiple-range test. Student’s *t*-test was used to evaluate the significance of differences between two groups of observations. *p* < 0.05 was considered statistically significant.

## Results

### High Glucose Treatment Causes Instant Membrane Repair Defect in MAECs

**FIGURE 1:**
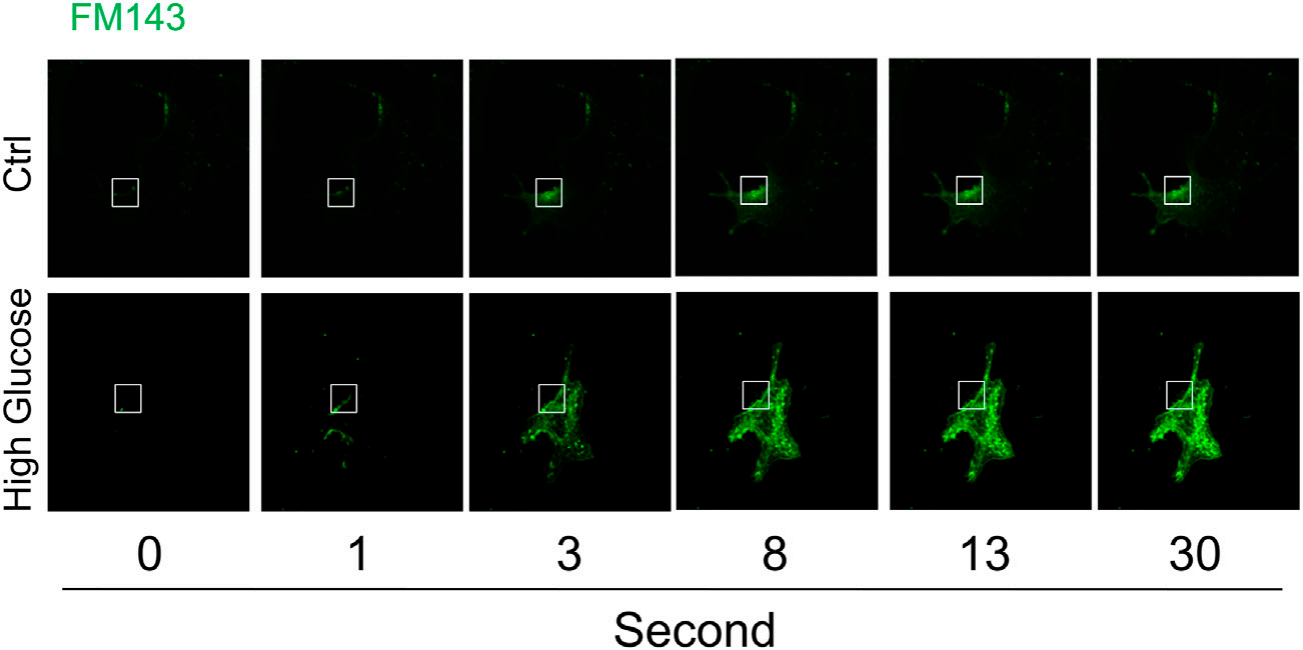
High glucose impairs instant membrane repair in MAECs. MAECs isolated from wild-type mice were incubated in the absence or presence of high glucose (38 mM) for 48 h and then the cells were subjected to laser wounding in the presence of lipophilic dye FM1-43. The wounded MAECs were bathed in normal Hank’s solution (with Ca^2+^). The fluorescence images of wounded cells were acquired. Representative images show the FM1-43 fluorescence at 0, 1, 3, 8, 13, and 30 s post laser-induced injury in cells under control or high glucose condition (the open box indicates the wounding site) (*n* = 5).

The time-lapse imaging of lipophilic dye FM1-43 was used to analyze instant membrane repair after laser-induced wounding in the plasma membrane in cells under normal control or high glucose condition. As shown in Figure 1, FM1-43 fluorescence was moderately increased in a time-dependent manner and mostly restricted to the plasma membrane in cells under normal control condition, which indicates that plasma membrane rapidly reseals after membrane injury and therefore such membrane repair through ICMR machinery limits the influx of lipophilic dye FM1-43. In contrast, the increase in FM1-43 fluorescence was more pronounced in the cells under high glucose condition suggesting that high glucose treatment causes a failure of membrane repair through ICMR machinery and thereby an uncontrolled influx of FM1-43 in these cells.

### Effects of Ca^2+^ and Death Receptor Stimulation on Instant Membrane Repair in MAECs

**FIGURE 2:**
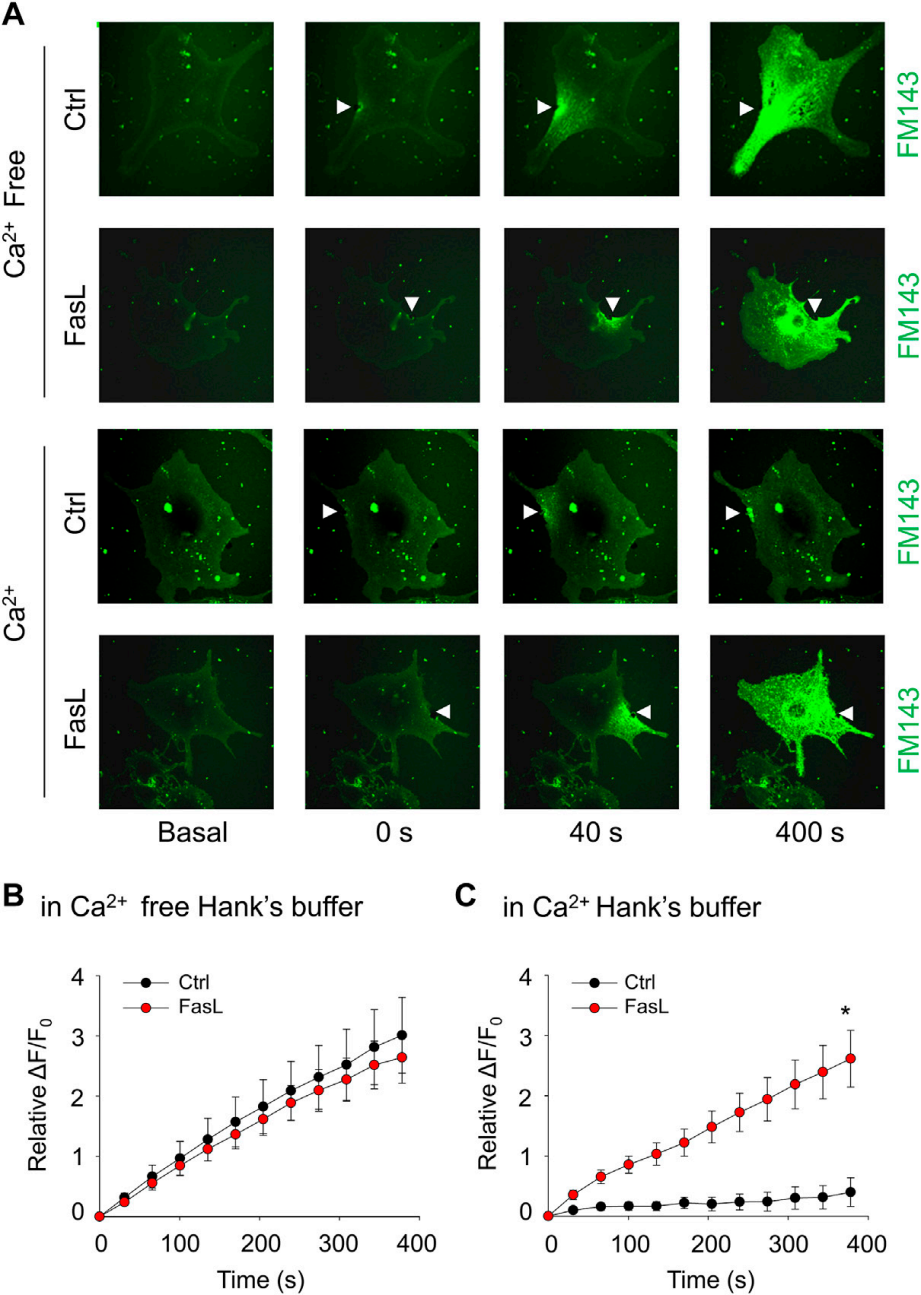
FasL stimulation prevents Ca^2+^-dependent membrane repair in MAECs. MAECs isolated from wild-type mice were pretreated with FasL (10 ng/ml) for 6 h and then subjected to laser wounding in the absence or presence of extracellular Ca^2+^. **(A)** Representative FM1-43 images were taken before injury (basal) and at 0, 40, and 400 s post laser post laser-induced injury in cells (arrowheads indicates wounding site). **(B)** and **(C)** Summarized data show the effects of Ca^2+^ and FasL on the FM1-43 fluorescence intensity in MAECs post laser-induced injury. *p < 0.05 vs. FasL (*n* = 5).

The plasma membrane lesions cause Ca^2+^ influx that triggers a membrane repairing mechanism involving Ca^2+^-regulated exocytosis (Idone et al., 2008). We next examined whether ICMR machinery in MAECs requires the presence of extracellular Ca^2+^. As shown in Figures 2A,B, the FM1-43 fluorescence was markedly increased in both control and FasL-treated MAECs when these cells were bathed in Ca^2+^-free Hank’s solution. Therefore, it is suggested that the presence of extracellular Ca^2+^ is a pre-requisite for initiating ICMR machinery upon membrane injury. Our data also demonstrated that FM1-43 was massively increased in FasL-pretreated MAECs in normal Hanks’ solution suggesting that stimulation of death receptor by FasL disrupts instant membrane repair machinery.

### ASM-Ceramide Pathway Is Involved in Instant Membrane Repair in MAECs

**FIGURE 3:**
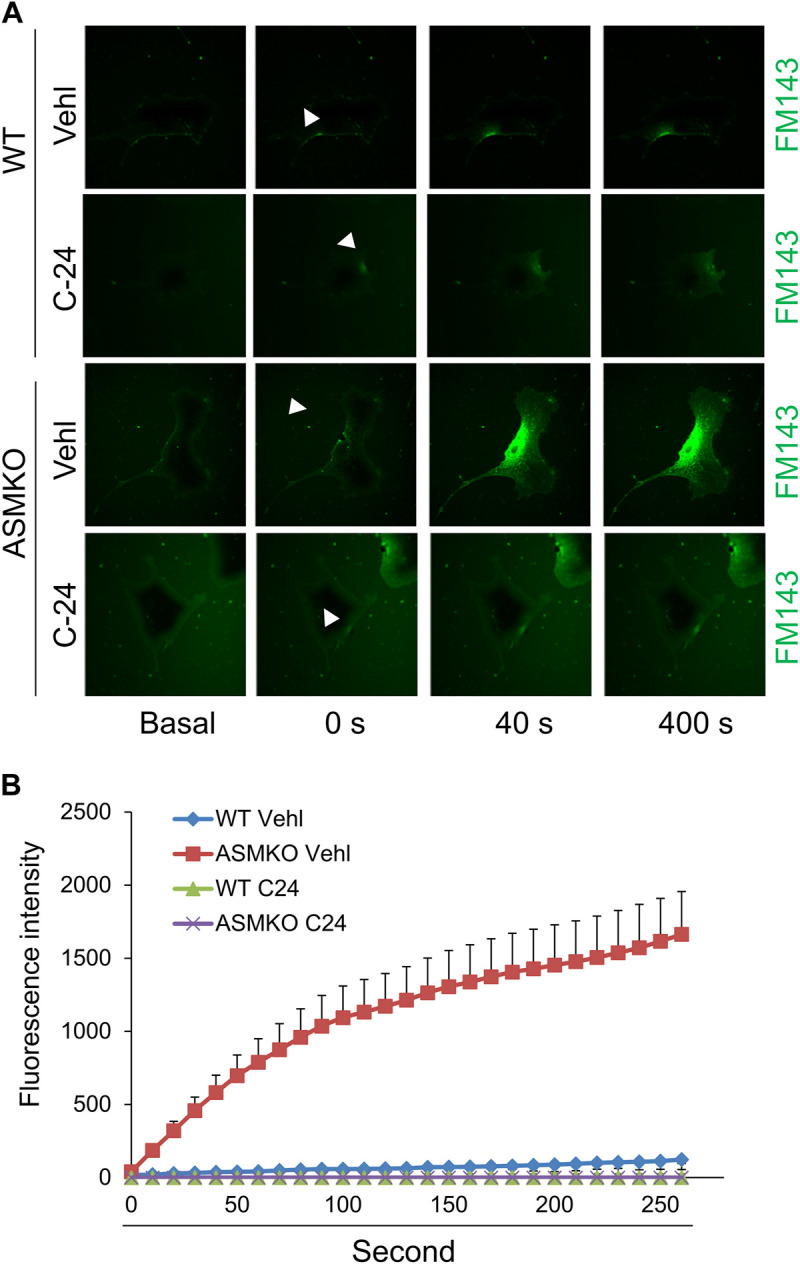
ASM-ceramide pathway is involved in instant membrane repair in MAECs. The ASM^+/+^ (WT) or ASM^−/−^ (ASMKO) MAECs were pretreated with or without C-24 ceramide (10 μM) for 30 min and then subjected to laser wounding in normal Hank’s solution (with Ca^2+^). **(A)** Representative FM1-43 images were taken before injury (basal) and at 0, 40, and 400 s post laser post laser-induced injury in cells (arrowheads indicates wounding site). **(B)** Summarized data show the effects of ASM gene deletion or C24 ceramide on the FM1-43 fluorescence intensity in MAECs post laser-induced injury (*n* = 5).

**FIGURE 4:**
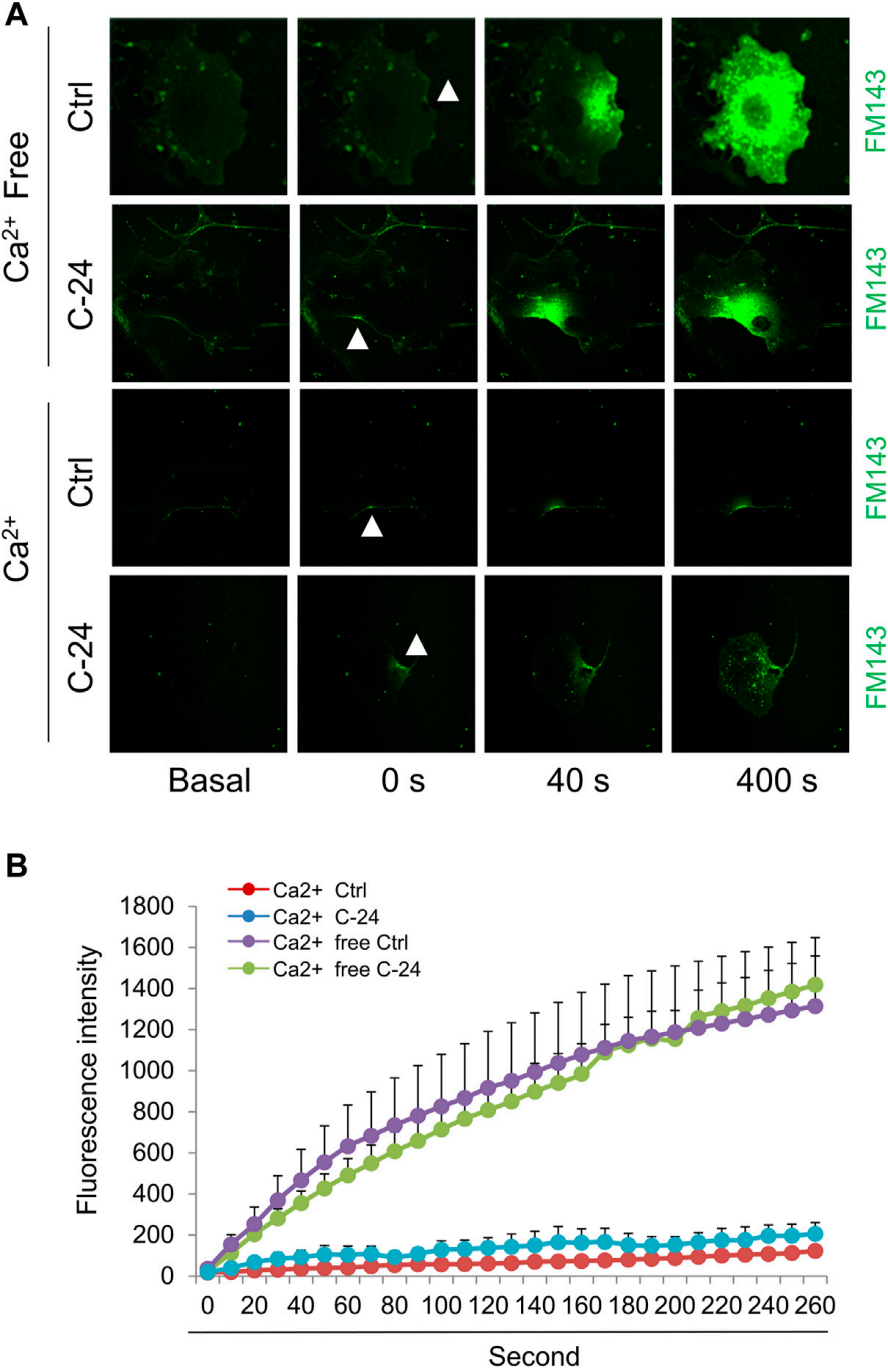
Ceramide initiates instant membrane repair in MAECs independent of extracellular Ca^2+^. The wild-type MAECs were pretreated with or without C-24 ceramide (10 μM) for 30 min and then subjected to laser wounding in the absence or presence of extracellular Ca^2+^. **(A)** Representative FM1-43 images were taken before injury (basal) and at 0, 40, and 400 s post laser post laser-induced injury in cells (arrowheads indicates wounding site). **(B)** Summarized data show the effects of C24 ceramide on the FM1-43 fluorescence intensity in MAECs post laser-induced injury in the absence or presence of extracellular Ca^2+^ (*n* = 5).

Ca^2+^-triggered exocytosis of lysosomes following plasma membrane intrusion releases lysosomal ASM that remodels plasma membrane by forming inward-budding microdomains, which may promotes lesion removal via endocytosis (Tam et al., 2010; Li et al., 2012). In MAECs isolated from ASMKO mice, we observed a significant intracellular staining of FM1-43 following laser induced wounding compared to that in WT cells (Figures 3A,B). Moreover, C-24 ceramide treatment blocked membrane injury-induced FM1-43 staining in ASMKO cells (Figures 3A,B), which indicates that C-24 ceramide could rescue membrane repair in ASMKO cells. In contrast, under Ca^2+^ free condition, C-24 ceramide could not rescue the instant membrane repair in MAECs (Figures 4A,B). Thus, these data suggest that in MAECs, ASM-ceramide signaling pathway in involved in extracellular Ca^2+^ influx-dependent ICMR machinery.

### Sphingomyelin and Ceramide Prevent High Glucose-Induced Defect in Instant Membrane Repair in MAECs

**FIGURE 5:**
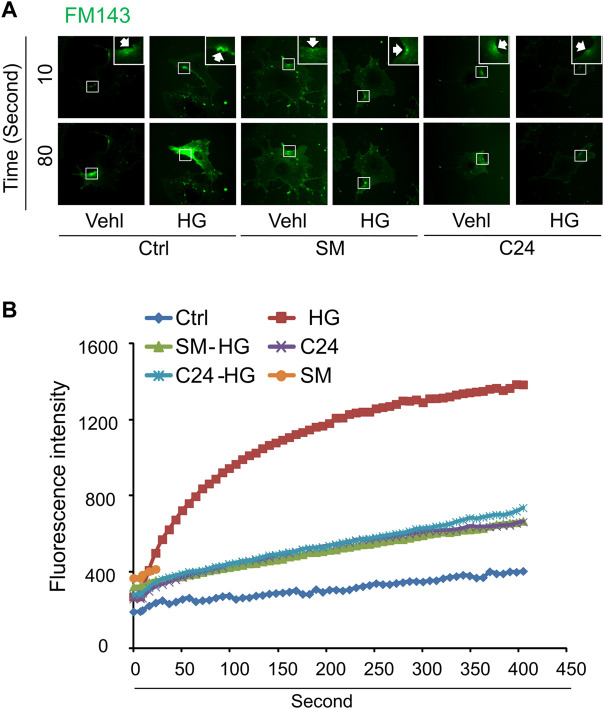
Sphingomyelin and ceramide prevents high glucose-induced instant membrane repair defect in MAECs. MAECs isolated from wild-type mice were treated with high glucose for 48 h and then subjected to laser wounding in normal Hank’s solution (with Ca^2+^). Sphingomyelin (SM 20 μM) or C-24 ceramide (10 μM) were added 30 min prior to laser wounding. **(A)** Representative FM1-43 images were taken before injury (basal) and at 10 and 80 s post laser-induced injury in cells (arrowheads indicates wounding site). **(B)** Summarized data show the effects of sphingomyelin and C24 ceramide on the FM1-43 fluorescence intensity in MAECs post 18 laser-induced injury in the absence or presence of high glucose (*n* = 5).

We next determined whether supplementation of exogenous sphingomyelin or ceramide could rescue the ICMR machinery in high glucose-treated MAECs. As shown in Figures 5A,B, cells treated with sphingomyelin or C-24 ceramide had no effects on FM1-43 dye uptake under normal condition. However, high glucose-induced uptake of FM-143 was largely prevented in cells pretreated with either sphingomyelin or C-24 ceramide. These data suggest that high glucose treatment may decrease the availability of the sphingomyelin pool for ASM-mediated ceramide production that contributes to ICMR.

### High Glucose Decreases Annexin A5 Expression and its Association With Ceramide in the Plasma Membrane of MAECs

**FIGURE 6:**
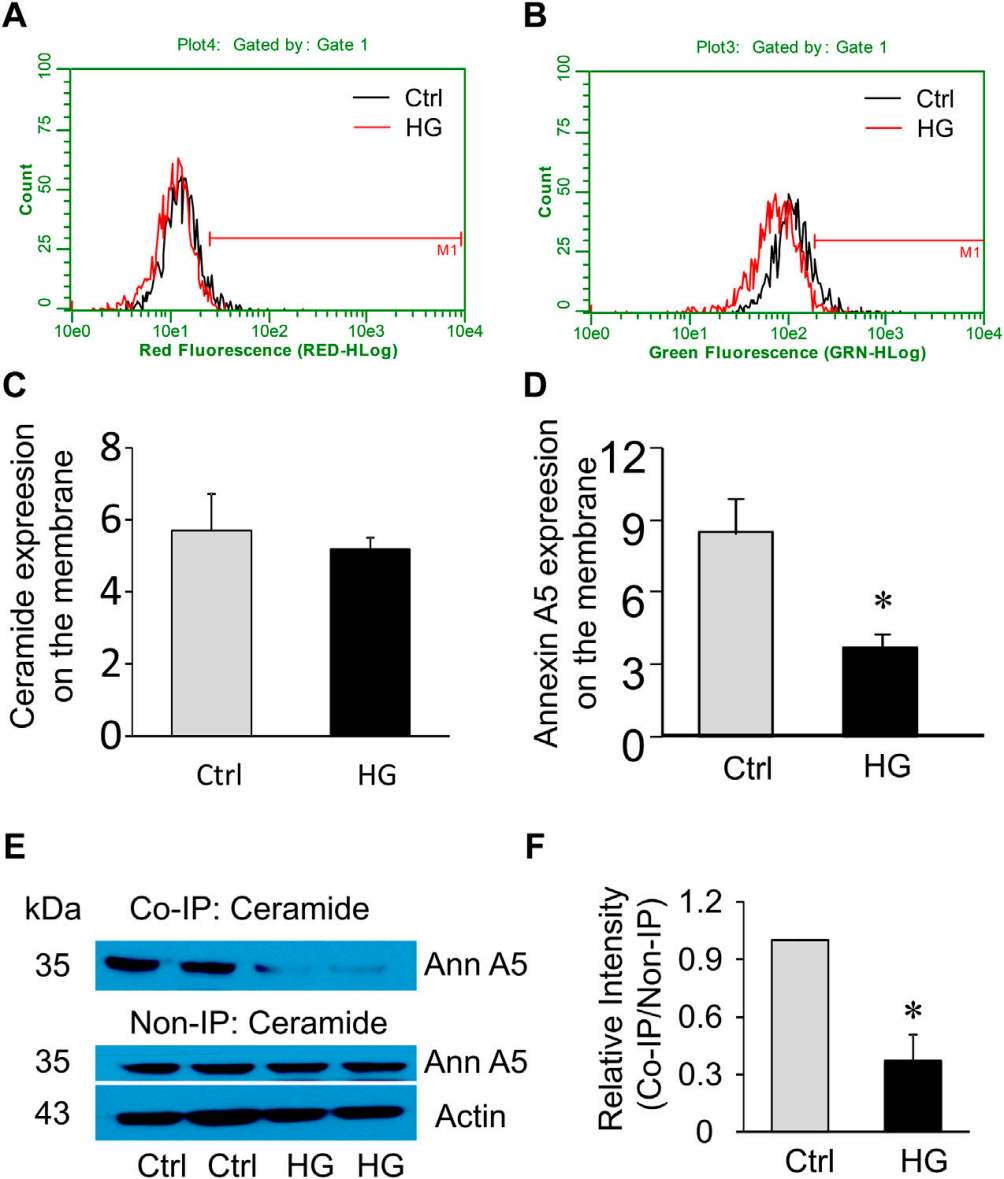
Effects of high glucose on surface annexin A5 and ceramide levels in MAECs. MAECs isolated from wild-type mice were treated with high glucose for 48 h and then were stained with FITC-conjugated antibodies against ceramide or annexin A5 following by flow cytometric analysis. **(A)** Flow cytometry analysis of surface ceramide level. **(B)** Flow cytometry analysis of surface expression of annexin A5. **(C,D)** Summarized data show the effects of high glucose on the mean fluorescence intensities for surface staining of ceramide or annexin A5. **(E)** Western blot analysis of annexin A5 protein in microsomes that was pulled down by ceramide antibodies and in whole cell lysates. **(F)**: Quantification represents the level of annexin A5 in the indicated immunoprecipitates. Ctrl: control; HG: High Glucose, *p < 0.05 vs. control (*n* = 5).

Extracellular Ca^2+^ influx triggers the recruitment and aggregation of annexin A5 to the membrane lesion in plasma membrane repair (Bouter et al., 2011; Carmeille et al., 2015). We sought to examine the effects of high glucose treatment on ceramide and annexin A5 expression levels in the plasma membrane of MAECs. High glucose treatment had no effects on ceramide level in the plasma membrane (Figures 6A,C) but markedly reduced the surface expression of annexin A5 (Figures 6B,D). High glucose treatment also decreased annexin A5 expression in co-immunoprecipitates pulled down together with ceramide; however, the total annexin A5 expression was not altered by high glucose (Figures 6E,F). Together, these results suggest that high glucose treatment disrupts the ceramide-annexin A5 association and thereby leads to intracellular redistribution of annexin A5 and reduction of surface expression of annexin A5 in MAECs.

### Decreased Ceramide-annexin A5 Association in Mouse Coronary Arterial Endothelium in Mice With Streptozotocin-Induced Diabetes

**FIGURE 7:**
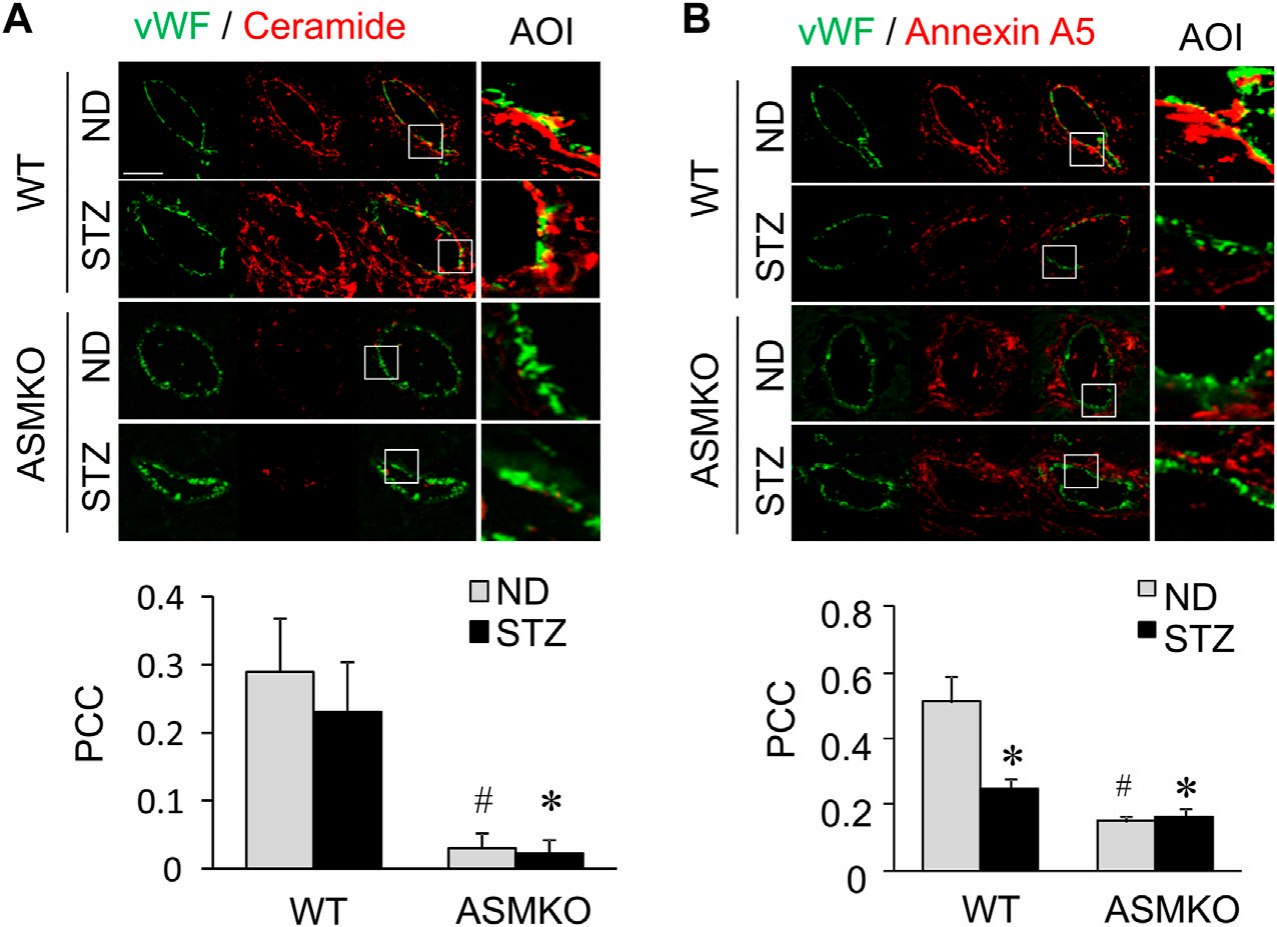
Effects of hyperglycemia on annexin A5 and ceramide in coronary arterial endothelium of mice with streptozotocin-induced diabetes. ASM^+/+^ (WT) or ASM^−/−^ (ASMKO) mice were administrated without (ND) or without streptozotocin (STZ). Frozen sections of mouse hearts were stained with Alexa488-anti-vWF and Alexa555-anti-ceramide or Alexa555-anti-annexin A5. **(A)** Representative fluorescent images show the ceramide (red) in the endothelium visualized by vWF staining (green). The summarized data show the co-localization coefficiency indicating the relative ratio of ceramide over vWF (*n* = 5–7). **(B)**: Representative fluorescent images show the expression of annexin A5 (red) in the endothelium visualized by vWF staining (green). The summarized data show the co-localization coefficiency indicating the relative ratio of annexin A5 over vWF (*n* = 5–8). *p < 0.05 vs. WT ND; #*p* < 0.05 vs. WT with STZ. Enlarged images of area of interest (AOI) in merged images are shown. Scale bar = 50 μm.

To examine the effects of hyperglycemia on the association between ceramide level and annexin A5 expression in the coronary endothelium, we analyze the co-localization of ceramide or annexin A5 to endothelial marker vWF in coronary arteries of mice with streptozotocin-induced diabetes. As shown in Figures 7A,B, in coronary arteries of ASM^+/+^ (WT) mice, streptozotocin treatment did not change the co-localization of ceramide with vWF (yellow fluorescence staining), whereas the co-localization of annexin A5 with vWF was markedly decreased in these arteries. These data suggests that hyperglycemia may disrupt the ceramide-annexin A5 association in the endothelium *in vivo*. Our data further demonstrate that, in mice under either control condition or with streptozotocin-induced diabetes, ASM gene deletion causes a decrease in the co-localization of ceramide with vWF, which correlates well with a concomitant decrease in the co-localization of annexin A5 with vWF. Therefore, our dada indicates that ASM-mediated ceramide pathway is essential for ceramide-annexin A5 association *in vivo*.

### Defective Membrane Repair in Coronary Arterial Endothelium Mice With Streptozotocin-Induced Diabetes

**FIGURE 8:**
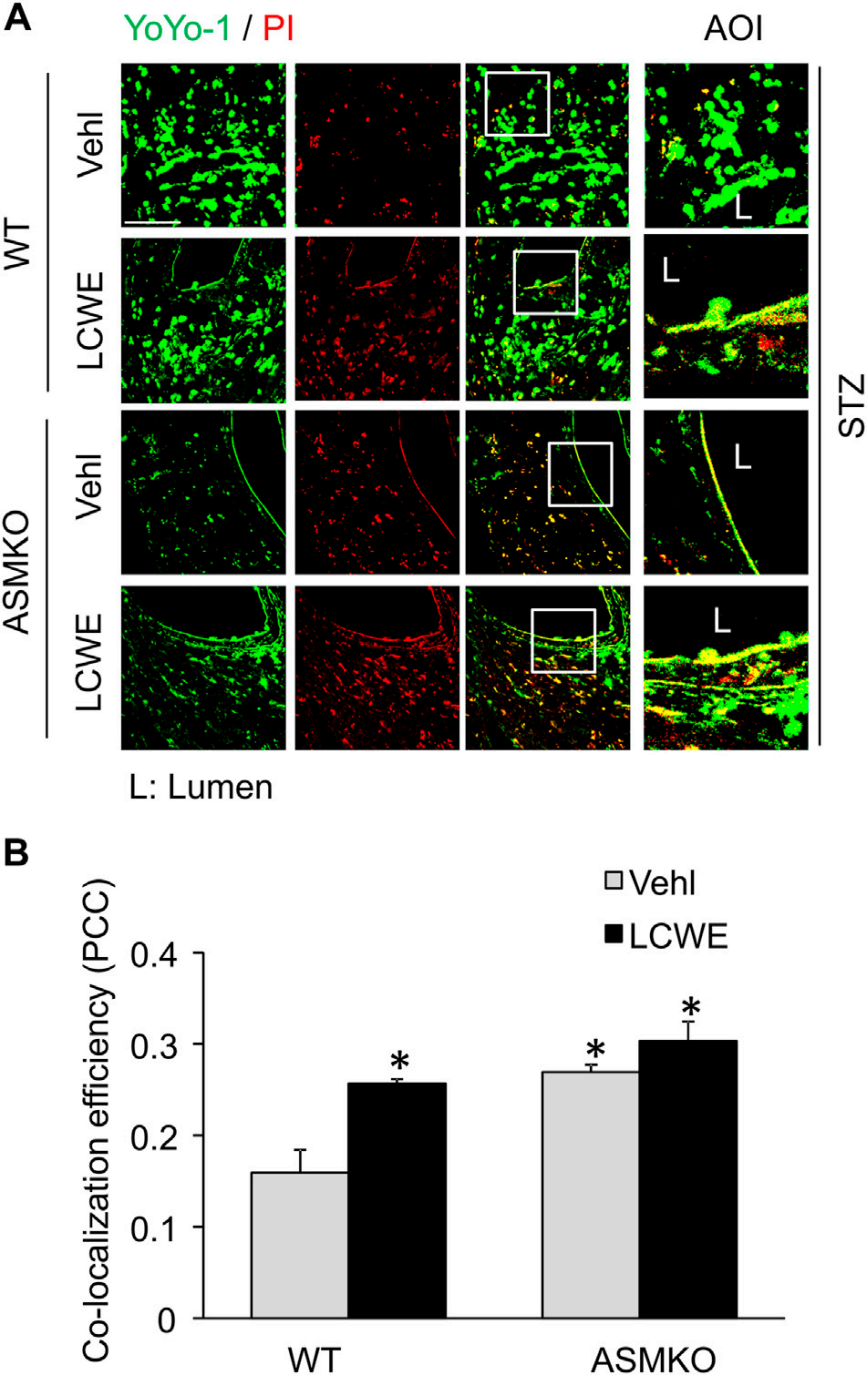
Defective membrane repair in coronary arterial endothelium in mice with streptozotocin-induced diabetes. ASM^+/+^ (WT) or ASM^−/−^ (ASMKO) mice were administrated with streptozotocin (STZ) and then treated with or without LCWE. YOYO-1 (Green) and propidium iodide (PI) (Red) was intravenously injected *via* inguinal veins to characterize endothelial cells with membrane repair defects. The frozen sections of mouse hearts were used for confocal microscopic analysis. **(A)** Representative fluorescent images show the colocalization of YOYO-1 and PI (*n* = 5–7). **(B)**: The summarized data show the co-localization coefficiency indicating the relative ratio of YOYO-1 over PI. *p < 0.05 vs. WT; #*p* < 0.05 vs. ASM^+/+^ LCWE. Enlarged images of area of interest (AOI) in merged images are shown. Scale bar = 50 μm.

We next examined if hyperglycemia and/or ASM deficiency causes defective plasma membrane repair machinery in the endothelium *in vivo*. LCWE is a commonly used bacterial extract to produce arteritis and a classical cell membrane hole producing agent (Chen et al., 2015a; Chen et al., 2016b). LCWE induces membrane injury that can be rapidly resealed in cultured endothelial cells (Chen et al., 2016b). Here, we determined whether the endothelial cell membrane fails to reseal in response to LCWE in coronary arteries of streptozotocin-induced diabetic mice. The endothelial cell membrane resealing *in vivo* was analyzed by a sequential staining of cells with YOYO-1, a membrane-impermeable dye with green florescence, and PI, a nuclear dye with red florescence. YOYO-1^+^/PI^+^ cells are considered as the cells with plasmalemma damage due to defective membrane resealing. As shown in Figures 8A,B, in ASM^+/+^ (WT) diabetic mice, LCWE treatment markedly increased the co-localization of YOYO-1 and PI in the perivascular area of coronary arteries. Further, control and LCWE-treated ASM^−/−^ (ASMKO) diabetic mice have similar levels of YOYO-1/PI co-localization in coronary endothelium when compared to that of LCWE-treated ASM^+/+^ diabetic mice. These data suggest that the endothelium of diabetic mice have defective membrane repair machinery upon membrane injurious factors that leads to exaggerated plasmalemma damage.

## Discussion

Our study identifies a novel effect of high glucose on endothelial cell pathobiology. We demonstrated that high glucose impairs instant membrane resealing, which is attributed to decreased association of membrane ceramide with annexin A5. Further, endothelial cells in coronary arteries of diabetic mice exhibit failed membrane resealing in response to membrane injury *in vivo*.

The present study first determined whether high glucose treatment causes a defect in the plasma membrane resealing in endothelial cells by observing the influx of lipophilic dye FM1-43. Our confocal microscopic data demonstrated that upon laser-induced wounding in the plasma membrane, the control cells rapidly repair the membrane as shown by limited FM1-43 staining, whereas high glucose-treated cells fails to repair the laser-induced wounding as shown by time-dependent increase in FM1-43 staining (Figure 1). Similar to our finding, a previous study also demonstrated that high glucose induced membrane repair defect in BSC-1 cells (Howard et al., 2011a). Previous studies suggest that instant cell membrane resealing (ICMR) is an active process that requires extracellular Ca^2+^, intracellular vesicles, and a step of Ca^2+^-dependent exocytosis (Zhou et al., 2008; Howard et al., 2011b; Bouter et al., 2011; Cheng et al., 2015). Consistently, we demonstrated that laser-induced wounding triggers an extracellular Ca^2+^-dependent membrane resealing pathway in endothelial cells (Figure 2). To our knowledge, these results provide the first evidence that high glucose leads to failure of Ca^2+^-mediated membrane resealing in endothelial cells in response to membrane injury.

Recent studies highlight a critical role of ASM-ceramide signaling pathway in cell membrane repair during cell injury (Babiychuk et al., 2008; Tam et al., 2010). It has been proposed that instant membrane resealing mechanism is initiated by extracellular Ca^2+^ influx through plasma injurious sites, which triggers intracellular vesicular trafficking to the plasma membrane, exocytosis and delivery of lysosomal ASM to the cell surface. ASM hydrolyzes sphingomyelin in the outer leaflet membrane into ceramide, a lipid that tends to coalesce on membranes forming inward-budding that may facilitate removal of injurious membrane via endocytosis. Consistently, our data demonstrated that membrane resealing is impaired following injury in ASM-deficient cells, whereas this impairment can be rescued by supplementation of ceramide (Figure 3). It should be noted that ceramide cannot rescue the membrane repair in the absence of extracellular Ca^2+^ (Figure 4). Therefore, these data suggest that the ICMR machinery is more complex than a simple ceramide-mediated inward-budding model. In response to extracellular Ca^2+^ influx, both ceramide-dependent and–independent signaling pathways are mobilized to act synergistically for efficient membrane resealing.

Previous studies have demonstrated that high glucose increased lysosome exocytosis, ceramide production, and the clustering of sphingomyelin- and cholesterol-enriched microdomains or membrane rafts (MRs) in human vascular endothelial cells (Bao et al., 2012; Wang et al., 2013). Activation of ASM-ceramide pathway was observed in diabetic animals and patients (Opreanu et al., 2011; Kady et al., 2017) and in human vascular endothelial cells under high glucose (Bao et al., 2012; Jiang et al., 2019). Ceramide can spontaneously fuse small MRs to promote MR clustering (Zhang Y. et al., 2009). Therefore, under diabetic and high glucose condition, activation of ASM may produce ceramide resulting in MR clustering in plasma membrane of endothelial cells. The present study showed that FasL, a strong inducer of MR cluster formation in endothelial cells (Zhang et al., 2006; Zhang S. et al., 2009; Bao et al., 2010), significantly attenuated the cell membrane resealing when the cells are exposed to laser-induced injury (Figure 2). Further, exogenous sphingomyelin or ceramide could rescue the membrane repair in high glucose-treated cells (Figure 5). Based on these observations, we proposed a model that the distribution pattern of sphingomyelin-enriched MRs in the plasma membrane correlates with the capability of ceramide-mediated membrane repair machinery. Under normal condition, MRs are evenly distributed through the membrane that provides the sphingomyelin pool for ASM-mediated ceramide production around the membrane lesion. Under high glucose condition, increased MR clustering in the plasma membrane might decrease the availability of the sphingomyelin pool around the lesion area leading to attenuated ceramide-mediated membrane resealing.

The present study further identifies a potential role of ceramide in modulating surface expression of annexin A5 in endothelial cells. Annexin A5 has been shown to play a central role in the membrane repair machinery by forming a two-dimensional bandage at the level of torn membrane edges, thus preventing the expansion of membrane wound and facilitating the final steps of membrane resealing (Bouter et al., 2011). Our *in vitro* data suggest that ceramides or ceramide associated molecules interact with annexin A5, whereas this association is disrupted by high glucose treatment. The present study further demonstrated that such ceramide-annexin A5 association is attenuated in the coronary arterial endothelium of diabetic mice or mice with ASM gene deletion. Therefore, it is proposed that the hyperglycemia decreases sphingomyelin availability and thus ceramide production around lesion area, which in turn disrupts the association of ceramide with annexin A5. Such disruption of ceramide-annexin A5 decreases surface expression of annexin A5 in the plasma membrane, which leads to an impaired aggregation of annexin A5 around lesion area and failed membrane repair machinery.

Lastly, we examined the impairment of ceramide-mediated membrane resealing in coronary arteries of diabetic mice. The present study showed that endothelial cells could not recover from laser-induced membrane injury when these cells are cultured under high glucose condition. Consistent with this *in vitro* finding, increased number of damaged cells with unrepaired membranes was observed in diabetic mice that were challenged with membrane puncturing agent LCWE. These data implicate that hyperglycemia causes defective membrane resealing in endothelial cells and thereby these cells are more vulnerable to membrane damage when they are exposed to a secondary insult (e.g., membrane injuring factors). Interestingly, the diabetic mice with ASM gene deletion exhibited increased number of damaged endothelial cells even in the absence of LCWE. It seems that endothelial cells from ASM-deficient diabetic mice are unable to be recovered from spontaneous membrane damages, which further highlights an essential role of ASM-ceramide pathway for membrane repair machinery in endothelial cells. A limitation of the study is a lack of recuse experiments *in vivo* to restore the sphingomyelin or ceramide availability in the plasma membrane in diabetic mice. The supplementation of ceramide or sphingomyelin *in vivo* is challenging as these lipids may largely be incorporated into membrane of cells or lipoproteins in the blood. Sphingomyelin synthase (SMS) is the enzyme for the *de novo* synthesis of sphingomyelin in the Golgi, which is then transported to plasma membrane. Future study will determine the effects of endothelial cell-specific overexpression of SMS gene on membrane resealing defects in diabetic mice.

In summary, the present study demonstrated that hyperglycemia impairs ASM-ceramide-mediated membrane repair machinery in endothelial cells *in vitro* and *in vivo*. This hyperglycemia-induced failure in membrane resealing may increase the vulnerability of endothelial cells to cell membrane damages when they are exposed to other pathological stimuli or danger factors. Our data provide novel insights into the pathogenic mechanism for hyperglycemia-induced endothelial dysfunction and vasculopathy.

## Data Availability

The raw data supporting the conclusions of this article will be made available by the authors, without undue reservation.

## References

[B1] Abreu-Blanco M. T. Watts J. J. Verboon J. M. Parkhurst S. M. (2012). Cytoskeleton Responses in Wound Repair. Cell. Mol. Life Sci. 69, 2469–2483. 10.1007/s00018-012-0928-2 22349211 PMC3388155

[B2] Babiychuk E. B. Monastyrskaya K. Draeger A. (2008). Fluorescent Annexin A1 Reveals Dynamics of Ceramide Platforms in Living Cells. Traffic 9, 1757–1775. 10.1111/j.1600-0854.2008.00800.x 18694456

[B3] Bansal D. Miyake K. Vogel S. S. Groh S. Chen C.-C. Williamson R. (2003). Defective Membrane Repair in Dysferlin-Deficient Muscular Dystrophy. Nature 423, 168–172. 10.1038/nature01573 12736685

[B4] Bao J.-X. Chang H. Lv Y.-G. Yu J.-W. Bai Y.-G. Liu H. (2012). Lysosome-membrane Fusion Mediated Superoxide Production in Hyperglycaemia-Induced Endothelial Dysfunction. PLoS One 7, e30387. 10.1371/journal.pone.0030387 22253932 PMC3257261

[B5] Bao J.-X. Xia M. Poklis J. L. Han W.-Q. Brimson C. Li P.-L. (2010). Triggering Role of Acid Sphingomyelinase in Endothelial Lysosome-Membrane Fusion and Dysfunction in Coronary Arteries. Am. J. Physiology-Heart Circulatory Physiology 298, H992–H1002. 10.1152/ajpheart.00958.2009 PMC2838547 20061541

[B6] Boini K. M. Xia M. Li C. Zhang C. Payne L. P. Abais J. M. (2011). Acid Sphingomyelinase Gene Deficiency Ameliorates the Hyperhomocysteinemia-Induced Glomerular Injury in Mice. Am. J. Pathology 179, 2210–2219. 10.1016/j.ajpath.2011.07.019 PMC3204029 21893018

[B7] Bouter A. Gounou C. Bérat R. Tan S. Gallois B. Granier T. (2011). Annexin-A5 Assembled into Two-Dimensional Arrays Promotes Cell Membrane Repair. Nat. Commun. 2, 270. 10.1038/ncomms1270 21468022 PMC3104517

[B8] Carmeille R. Degrelle S. A. Plawinski L. Bouvet F. Gounou C. Evain-Brion D. (2015). Annexin-A5 Promotes Membrane Resealing in Human Trophoblasts. Biochimica Biophysica Acta (BBA) - Mol. Cell Res. 1853, 2033–2044. 10.1016/j.bbamcr.2014.12.038 25595530

[B9] Chen Y. Yuan M. Xia M. Wang L. Zhang Y. Li P. L. (2016b). Instant Membrane Resealing in Nlrp3 Inflammmasome Activation of Endothelial Cells. Front. Biosci. (Landmark Ed. 21, 635–650. 10.2741/4411 26709796 PMC5507337

[B10] Chen Y. Li X. Boini K. M. Pitzer A. L. Gulbins E. Zhang Y. (2015a). Endothelial Nlrp3 Inflammasome Activation Associated with Lysosomal Destabilization during Coronary Arteritis. Biochimica Biophysica Acta (BBA) - Mol. Cell Res. 1853, 396–408. 10.1016/j.bbamcr.2014.11.012 PMC4289419 25450976

[B11] Chen Y. Pitzer A. L. Li X. Li P. L. Wang L. Zhang Y. (2015b). Instigation of Endothelial Nlrp3 Inflammasome by Adipokine Visfatin Promotes Inter‐endothelial Junction Disruption: Role of HMGB 1. J. Cell. Mol. Med. 19, 2715–2727. 10.1111/jcmm.12657 26293846 PMC4687695

[B12] Chen Y. Wang L. Pitzer A. L. Li X. Li P.-L. Zhang Y. (2016a). Contribution of Redox-dependent Activation of Endothelial Nlrp3 Inflammasomes to Hyperglycemia-Induced Endothelial Dysfunction. J. Mol. Med. 94, 1335–1347. 10.1007/s00109-016-1481-5 27783111 PMC5512566

[B13] Cheng X. Zhang X. Yu L. Xu H. (2015). Calcium Signaling in Membrane Repair. Seminars Cell & Dev. Biol. 45, 24–31. 10.1016/j.semcdb.2015.10.031 PMC4681278 26519113

[B14] Draeger A. Schoenauer R. Atanassoff A. P. Wolfmeier H. Babiychuk E. B. (2014). Dealing with Damage: Plasma Membrane Repair Mechanisms. Biochimie 107, 66–72. 10.1016/j.biochi.2014.08.008 25183513

[B15] Horinouchi K. Erlich S. Perl D. P. Ferlinz K. Bisgaier C. L. Sandhoff K. (1995). Acid Sphingomyelinase Deficient Mice: a Model of Types A and B Niemann-Pick Disease. Nat. Genet. 10, 288–293. 10.1038/ng0795-288 7670466

[B16] Howard A. C. Mcneil A. K. Mcneil P. L. (2011a). Promotion of Plasma Membrane Repair by Vitamin E. Nat. Commun. 2, 597. 10.1038/ncomms1594 22186893 PMC3247818

[B17] Howard A. C. Mcneil A. K. Xiong F. Xiong W.-C. Mcneil P. L. (2011b). A Novel Cellular Defect in Diabetes. Diabetes 60, 3034–3043. 10.2337/db11-0851 21940783 PMC3198060

[B18] Idone V. Tam C. Goss J. W. Toomre D. Pypaert M. Andrews N. W. (2008). Repair of Injured Plasma Membrane by Rapid Ca2+-dependent Endocytosis. J. Cell Biol. 180, 905–914. 10.1083/jcb.200708010 18316410 PMC2265401

[B19] Jiang M. Huang S. Duan W. Liu Q. Lei M. (2019). Inhibition of Acid Sphingomyelinase Activity Ameliorates Endothelial Dysfunction in Db/db Mice. Biosci. Rep. 39, BSR20182144. 10.1042/BSR20182144 30910852 PMC6481240

[B20] Jin S. Yi F. Zhang F. Poklis J. L. Li P.-L. (2008). Lysosomal Targeting and Trafficking of Acid Sphingomyelinase to Lipid Raft Platforms in Coronary Endothelial Cells. Atvb 28, 2056–2062. 10.1161/atvbaha.108.172478 PMC2668813 18772496

[B21] Kady N. Yan Y. Salazar T. Wang Q. Chakravarthy H. Huang C. (2017). Increase in Acid Sphingomyelinase Level in Human Retinal Endothelial Cells and CD34+ Circulating Angiogenic Cells Isolated from Diabetic Individuals Is Associated with Dysfunctional Retinal Vasculature and Vascular Repair Process in Diabetes. J. Clin. Lipidol. 11, 694–703. 10.1016/j.jacl.2017.03.007 28457994 PMC5492962

[B22] Kandula V. Kosuru R. Li H. Yan D. Zhu Q. Lian Q. (2016). Forkhead Box Transcription Factor 1: Role in the Pathogenesis of Diabetic Cardiomyopathy. Cardiovasc Diabetol. 15, 44. 10.1186/s12933-016-0361-1 26956801 PMC4784400

[B23] Laight D. W. Carrier M. J. Änggård E. E. (1999). Endothelial Cell Dysfunction and the Pathogenesis of Diabetic Macroangiopathy. Diabetes Metab. Res. Rev. 15, 274–282. 10.1002/(sici)1520-7560(199907/08)15:4<274::aid-dmrr46>3.0.co;2-g 10495476

[B24] Li X. Gulbins E. Zhang Y. (2012). Oxidative Stress Triggers Ca2+-dependent Lysosome Trafficking and Activation of Acid Sphingomyelinase. Cell Physiol. Biochem. 30, 815–826. 10.1159/000341460 22890197 PMC3777434

[B25] Li X. Han W.-Q. Boini K. M. Xia M. Zhang Y. Li P.-L. (2013). TRAIL Death Receptor 4 Signaling via Lysosome Fusion and Membrane Raft Clustering in Coronary Arterial Endothelial Cells: Evidence from ASM Knockout Mice. J. Mol. Med. 91, 25–36. 10.1007/s00109-012-0968-y 23108456 PMC3537912

[B26] Liye H. Lvyun Z. Guangyao S. Luping R. (2011). Investigation of Early Change of Endothelial Function and Related Factors in Individuals with Hyperglycemia. Diabetes Res. Clin. Pract. 92, 194–197. 10.1016/j.diabres.2011.01.018 21334758

[B27] Mattson M. P. Zhu H. Yu J. Kindy M. S. (2000). Presenilin-1 Mutation Increases Neuronal Vulnerability to Focal IschemiaIn Vivoand to Hypoxia and Glucose Deprivation in Cell Culture: Involvement of Perturbed Calcium Homeostasis. J. Neurosci. 20, 1358–1364. 10.1523/jneurosci.20-04-01358.2000 10662826 PMC6772370

[B28] Mcneil P. L. (2002). Repairing a Torn Cell Surface: Make Way, Lysosomes to the Rescue. J. Cell Sci. 115, 873–879. 10.1242/jcs.115.5.873 11870206

[B29] Mcneil P. L. Steinhardt R. A. (2003). Plasma Membrane Disruption: Repair, Prevention, Adaptation. Annu. Rev. Cell Dev. Biol. 19, 697–731. 10.1146/annurev.cellbio.19.111301.140101 14570587

[B30] Miller H. Castro-Gomes T. Corrotte M. Tam C. Maugel T. K. Andrews N. W. (2015). Lipid Raft-dependent Plasma Membrane Repair Interferes with the Activation of B Lymphocytes. J. Cell Biol. 211, 1193–1205. 10.1083/jcb.201505030 26694840 PMC4687878

[B31] Opreanu M. Tikhonenko M. Bozack S. Lydic T. A. Reid G. E. Mcsorley K. M. (2011). The Unconventional Role of Acid Sphingomyelinase in Regulation of Retinal Microangiopathy in Diabetic Human and Animal Models. Diabetes 60, 2370–2378. 10.2337/db10-0550 21771974 PMC3161322

[B32] Somanath S. Barg S. Marshall C. Silwood C. J. Turner M. D. (2009). High Extracellular Glucose Inhibits Exocytosis through Disruption of Syntaxin 1A-Containing Lipid Rafts. Biochem. Biophysical Res. Commun. 389, 241–246. 10.1016/j.bbrc.2009.08.126 19716806

[B33] Takase S. Matoba T. Nakashiro S. Mukai Y. Inoue S. Oi K. (2017). Ezetimibe in Combination with Statins Ameliorates Endothelial Dysfunction in Coronary Arteries after Stenting. Atvb 37, 350–358. 10.1161/atvbaha.116.308388 27932353

[B34] Tam C. Idone V. Devlin C. Fernandes M. C. Flannery A. He X. (2010). Exocytosis of Acid Sphingomyelinase by Wounded Cells Promotes Endocytosis and Plasma Membrane Repair. J. Cell Biol. 189, 1027–1038. 10.1083/jcb.201003053 20530211 PMC2886342

[B35] Wang A. Li C. Liao J. Dong M. Xiao Z. Lei M. (2013). Ceramide Mediates Inhibition of the Akt/eNOS Pathway by High Levels of Glucose in Human Vascular Endothelial Cells. J. Pediatr. Endocrinol. Metab. 26, 31–38. 10.1515/jpem-2012-0144 23457308

[B36] Wei Y.-M. Li X. Xiong J. Abais J. M. Xia M. Boini K. M. (2013). Attenuation by Statins of Membrane Raft-Redox Signaling in Coronary Arterial Endothelium. J. Pharmacol. Exp. Ther. 345, 170–179. 10.1124/jpet.112.201442 23435541 PMC3629800

[B37] Yu T. Jhun B. S. Yoon Y. (2011). High-glucose Stimulation Increases Reactive Oxygen Species Production through the Calcium and Mitogen-Activated Protein Kinase-Mediated Activation of Mitochondrial Fission. Antioxidants Redox Signal. 14, 425–437. 10.1089/ars.2010.3284 PMC3025178 20518702

[B38] Zhang A. Y. Yi F. Zhang G. Gulbins E. Li P.-L. (2006). Lipid Raft Clustering and Redox Signaling Platform Formation in Coronary Arterial Endothelial Cells. Hypertension 47, 74–80. 10.1161/01.hyp.0000196727.53300.62 16344372

[B39] Zhang P. Guan Y. Chen J. Li X. Mcconnell B. K. Zhou W. (2018). Contribution of p62/SQSTM1 to PDGF-BB-Induced Myofibroblast-like Phenotypic Transition in Vascular Smooth Muscle Cells Lacking Smpd1 Gene. Cell Death Dis. 9, 1145. 10.1038/s41419-018-1197-2 30451833 PMC6242941

[B40] Zhang S. Liu T. Liang H. Zhang H. Yan D. Wang N. (2009a). Lipid Rafts Uncouple Surface Expression of Transmembrane TNF-α from its Cytotoxicity Associated with ICAM-1 Clustering in Raji Cells. Mol. Immunol. 46, 1551–1560. 10.1016/j.molimm.2009.01.001 19203796

[B41] Zhang Y. Li X. Becker K. A. Gulbins E. (2009b). Ceramide-enriched Membrane Domains-Structure and Function. Biochimica Biophysica Acta (BBA) - Biomembr. 1788, 178–183. 10.1016/j.bbamem.2008.07.030 18786504

[B42] Zhang Y. Li X. Carpinteiro A. Goettel J. A. Soddemann M. Gulbins E. (2011). Kinase Suppressor of Ras-1 Protects against Pulmonary *Pseudomonas aeruginosa* Infections. Nat. Med. 17, 341–346. 10.1038/nm.2296 21297617

[B43] Zhou Y. Shi J. Cui J. Deng C. X. (2008). Effects of Extracellular Calcium on Cell Membrane Resealing in Sonoporation. J. Control. Release 126, 34–43. 10.1016/j.jconrel.2007.11.007 18158198 PMC2270413

[B44] Zimmet P. Alberti K. G. Magliano D. J. Bennett P. H. (2016). Diabetes Mellitus Statistics on Prevalence and Mortality: Facts and Fallacies. Nat. Rev. Endocrinol. 12, 616–622. 10.1038/nrendo.2016.105 27388988

